# Double-Sided Transparent TiO_2_ Nanotube/ITO Electrodes for Efficient CdS/CuInS_2_ Quantum Dot-Sensitized Solar Cells

**DOI:** 10.1186/s11671-016-1787-9

**Published:** 2017-01-04

**Authors:** Chong Chen, Lanyu Ling, Fumin Li

**Affiliations:** 1 Henan Key Laboratory of Photovoltaic Materials, Henan University, Kaifeng, 475004 People’s Republic of China; 2 School of Physics and Electronics, Henan University, Kaifeng, 475004 People’s Republic of China

## Abstract

In this paper, to improve the power conversion efficiencies (PCEs) of quantum dot-sensitized solar cells (QDSSCs) based on CdS-sensitized TiO_2_ nanotube (TNT) electrodes, two methods are employed on the basis of our previous work. First, by replacing the traditional single-sided working electrodes, double-sided transparent TNT/ITO (DTTO) electrodes are prepared to increase the loading amount of quantum dots (QDs) on the working electrodes. Second, to increase the light absorption of the CdS-sensitized DTTO electrodes and improve the efficiency of charge separation in CdS-sensitized QDSSCs, copper indium disulfide (CuInS_2_) is selected to cosensitize the DTTO electrodes with CdS, which has a complementary property of light absorption with CdS. The PCEs of QDSSCs based on these prepared QD-sensitized DTTO electrodes are measured. Our experimental results show that compared to those based on the CdS/DTTO electrodes without CuInS_2_, the PCEs of the QDSSCs based on CdS/CuInS_2_-sensitized DTTO electrode are significantly improved, which is mainly attributed to the increased light absorption and reduced charge recombination. Under simulated one-sun illumination, the best PCE of 1.42% is achieved for the QDSSCs based on CdS(10)/CuInS_2_/DTTO electrode, which is much higher than that (0.56%) of the QDSSCs based on CdS(10)/DTTO electrode.

## Background

Quantum dots-sensitized solar cells (QDSSCs) for converting solar energy directly to electricity have been attracting extensive interest for potential photovoltaic application [1–4]. In QDSSCs, the TiO_2_ is widely used as the working electrode due to its non toxicity, high stability, wide availability, and good electronic properties. However, it is known that the TiO_2_ mainly absorbs the ultraviolet light due to its large band gap of 3.2 eV. Therefore, various types of quantum dots (QDs) with different optical absorption properties, such as CdS [5–7], CdTe [8–10], CdSe [4, 11–14], PbS [15, 16], PbSe [17], and CuInS_2_ [3, 18], have been synthesized to sensitize the TiO_2_ in order to extend the light absorption of the TiO_2_ into the visible region. To further increase the light absorption of QD-sensitized TiO_2_, increasing the loading amount of QDs through the improvement of the TiO_2_ photoelectrode structures is an effective way. Recently, a novel electrode structure, i.e., double-sided transparent TiO_2_ nanotube/ITO (DTTO) photoelectrodes were successfully fabricated by our group to enhance light absorption of CdS QD-sensitized TiO_2_ photoelectrodes mainly due to the increase of CdS deposition amount [19], in which the TiO_2_ nanotube arrays are fabricated on the double-sided transparent ITO substrates. However, for these CdS QD-sensitized DTTO (CdS/DTTO) photoelectrodes, there is still a room for further improvement in light absorption capacity because the CdS/DTTO photoelectrodes mainly absorb visible light with wavelengths less than 550 nm [19]. Hence, for the CdS/DTTO photoelectrodes, there is a prevailing need to find a suitable semiconductor material with a lower band gap than that (2.4 eV) of CdS to harvest more light with wavelengths longer than 550 nm. Copper indium disulfide (CuInS_2_) with a narrow band gap of about 1.6 eV is used as the absorption materials in solar cells from its excellent electric and optical properties [3]. Our previous work has shown that the CuInS_2_ can be used as a co-sensitizer to extend the spectral response of CdS-sensitized TiO_2_ nanotubes (TNTs) on the Ti substrate into the 500–700 nm wavelength region [18]. Moreover, it has also found that the CuInS_2_ can reduce the charge recombination in CdS/CuInS_2_-sensitized TNTs/Ti electrode. Nevertheless, there is still an issue to be resolved. Due to the opaque Ti substrate, only the QDs deposited on one side of the TNTs/Ti electrode can absorb the sunlight. Obviously, the light-harvesting ability of the opaque TNTs/Ti photoelectrode should be weaker than that of the DTTO photoelectrode.

**Fig. 1:**
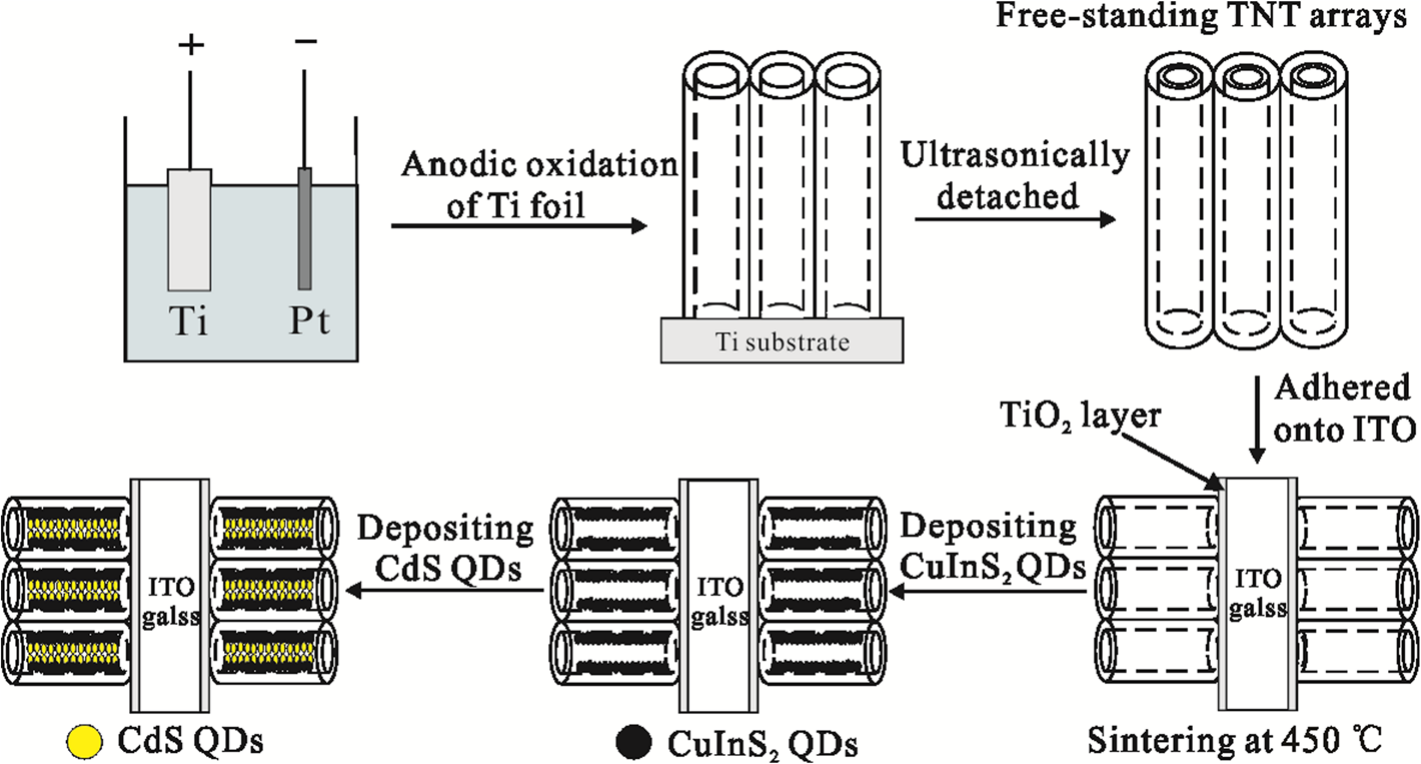
Synthetic strategy of the CdS/CuInS_2_/DTTO electrode

In this study, we expand our previous work [18, 19]. Considering the advantage of the DTTO photoelectrode in the light-harvesting ability and the complementary effect of CdS and CuInS_2_ on the light absorption, the CdS/CuInS_2_-co-sensitized DTTO photoelectrodes are prepared for the QDSSCs. The detailed synthetic strategy is illustrated in Fig. 1. The surface morphology, optical, and photoelectrochemical properties of as-prepared CdS/CuInS_2_/DTTO photoelectrodes are systematically studied. The obtained experimental results demonstrate that, compared to the CdS/DTTO photoelectrodes, the light absorption abilities and photoelectrochemical activities of the CdS/CuInS_2_/DTTO photoelectrodes are increased and the power conversion efficiencies (PCEs) of the QDSSCs based on the CdS/CuInS_2_/DTTO photoelectrodes are significantly enhanced.

## Methods

### Materials

Indium tin oxide (ITO, ≤15 Ω/∀) sheet glass, titanium foil (Ti, Sigma-Aldrich, 0.25-mm thickness, 99.7% purity), ammonium fluoride (NH_4_F, Sigma-Aldrich, 98 + %), ethylene glycol (Junsei Chemical Co, 99.0%), cadmium chloride (CdCl_2_, Kanto Chemical Co, 98.0%), indium choride (InCl_3_, Sigma-Aldrich, 99.999%), sodium sulfide nonahydrate (Na_2_S, Sigma-Aldrich, 98.0%), cupric chloride (CuCl_2_, Junsei Chemical co., Ltd, >97.0 + %), and Ti(OCH_2_CH_2_CH_2_CH_3_)_4_ (Ti(OBu)_4_, Sigma-Aldrich, 97%). All the materials were used directly without further purification.

### Synthesis of Double-Sided Transparent TNT/ITO Films

The TiO_2_ nanotube arrays (TNTs) were prepared by electrochemical anodization of the Ti foils. First, the electrolyte consisting of 0.5 wt% NH_4_F and 1.5 wt% distilled (DI) water in ethylene glycol (EG) was prepared. Before use, the electrolyte was stirred for 3 h at room temperature. After that, the cleaned Ti foils were anodized at a constant potential of 60 V in prepared electrolyte for 5 h in a two-electrode configuration with a platinum cathode [18]. Then, the formed TNTs were detached from the Ti substrate by intense ultrasonication in DI water. After that, the detached TNTs were adhered onto both sides of ITO glass with a drop of TiO_2_ sol. The process for the preparation of TiO_2_ sol containing Ti(OBu)_4_ and polyethylene glycol has been described in our previous work [19]. Finally, the as-prepared films were annealed at 450 °C for 2 h in air to crystallize the TiO_2_ tubes, after which the samples were naturally cooled down to room temperature to obtain the double-sided transparent TNT/ITO films (i.e., DTTO films).

### Synthesis of CdS/DTTO and CdS/CuInS_2_/DTTO Electrodes

CdS and CuInS_2_ QDs were deposited on the TNTs by CBD method and SILAR progress, respectively, as described in our previous papers [18, 20]. The CuInS_2_ was first deposited on the DTTO films by SILAR progress. The precursors are a 5 mM InCl_3_ aqueous solution, a 5 mM CuCl_2_ aqueous solution, and a 50 mM Na_2_S aqueous solution. The detailed one-cycle synthesis of CuInS_2_ can be obtained from previous publication [18]. In this study, the SILAR cycle was repeated two times.

CdS QDs were deposited on the DTTO and CuInS_2_/DTTO electrodes by CBD method. The precursors are a 50 mM Na_2_S aqueous solution and a 50 mM CdCl_2_ aqueous solution. The electrode was first dipped into 50 mM Na_2_S aqueous solution for 1 min, and then rinsed with DI water. After that, the electrode was dipped into 50 mM CdCl_2_ aqueous solution for another 1 min, and then rinsed again with DI water. Such a soaking and cleaning process is a typical CBD cycle of CdS deposition. The DTTO and CuInS_2_/DTTO electrodes after *n* cycles of CdS deposition are denoted as CdS(*n*)/DTTO and CdS(*n*)/CuInS_2_/DTTO, respectively.

### Characterization

The SEM images were recorded on a field-emission scanning electron microscopy (FESEM, FEI, Nova230). UV–vis absorption spectra were recorded using a UV–vis spectrophotometer (UV-2550, Shimadzu Corporation, Kyoto, Japan). Transmission electron microscope (TEM) analysis was done on a Tecnai G2 F30 TEM (FEI Company). Photoelectrochemical reactions of as-prepared samples were carried out in a 250-mL quartz cell, using a two-electrode configuration with the as-prepared samples as working electrode and a Pt counter electrode. A 3-m double-sided adhesive tape sandwiched between the work electrode and the Pt electrode is used to fix the distance between these two electrodes. The photocurrent–voltage characteristics of as-prepared samples with an effective surface area of 0.1 cm^−2^ were recorded using an electrochemical workstation (CHI660E, Shanghai Chenhua Instruments Co., Ltd., Shanghai, China) under simulated AM 1.5G illumination (100 mW cm^−2^) provided by a solar simulator equipped with a 500 W Xe lamp. The electrolyte was 1.0 M Na_2_S aqueous solution.

## Results and Discussion

**Fig. 2:**
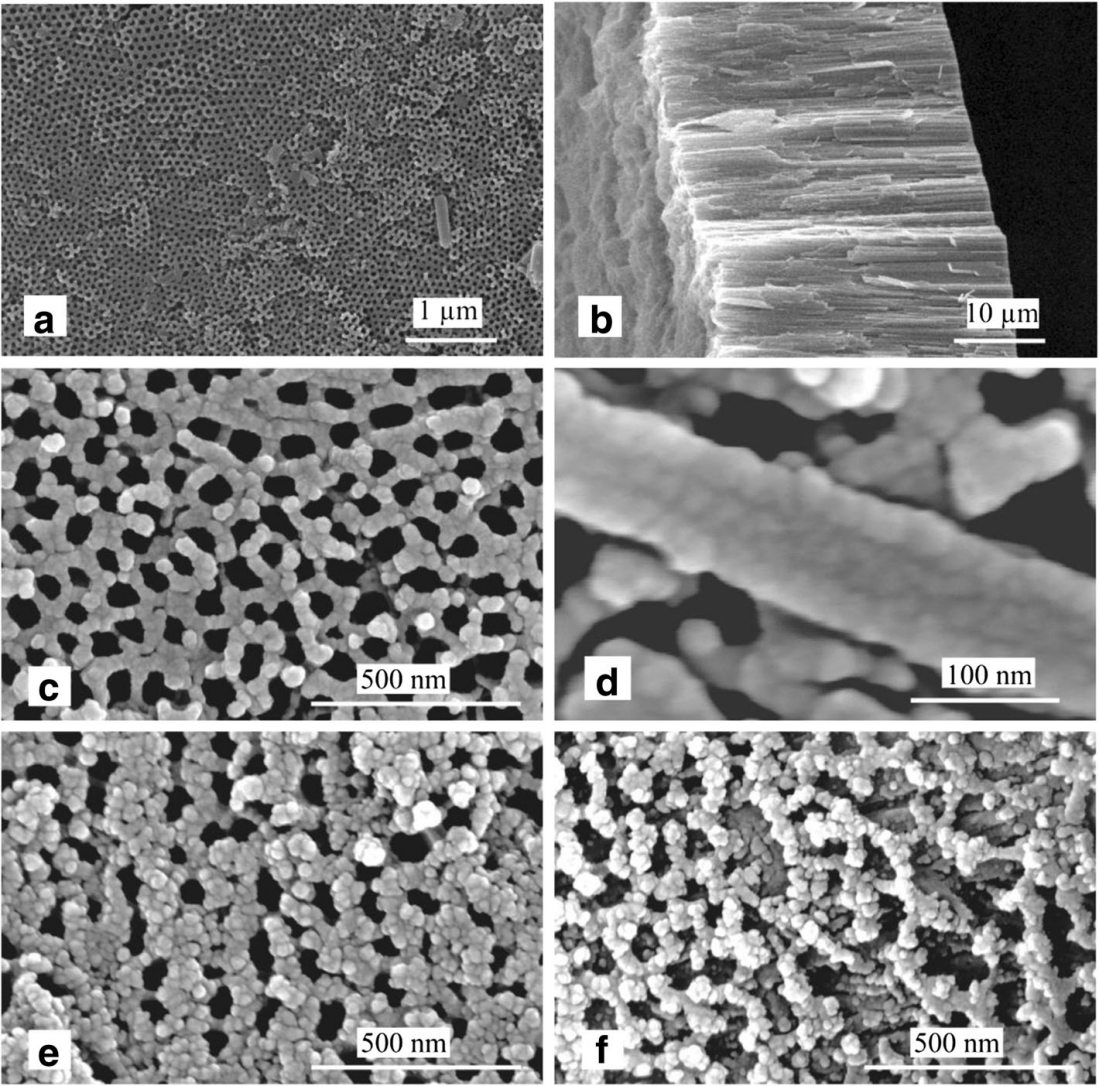
**a** A top-view SEM image of prepared DTTO film. **b** A cross-sectional view of the detached TNTs from Ti substrate. **c** A top-view image of the CuInS_2_/DTTO film. **d** A SEM image of one single CuInS_2_/TiO_2_ nanotube. Top-view images of as-prepared CdS(10)/CuInS_2_/DTTO (**e**) and CdS(15)/CuInS_2_/DTTO (**f**) films

Figure 2a, b shows the top-view SEM image of prepared DTTO film and the cross-section SEM image of the detached TNTs from Ti substrate, respectively. As shown in Fig. 2a, the highly ordered TNTs with an average inner diameter of ~100 nm and a wall thickness of ~20 nm are formed. From Fig. 2b, the TNTs with a length up to about 30 μm can be observed. Figure 2c displays the top-view image of the CuInS_2_/DTTO film. It can be observed from Fig. 2c that some CuInS_2_ nanoparticles were dispersed on the surface of CuInS_2_/DTTO film. Moreover, compared to the TiO_2_ nanotube in the DTTO film, the inner diameter of CuInS_2_/TiO_2_ nanotube decreased slightly due to the deposition of CuInS_2_. Figure 2d displays a SEM image of one single CuInS_2_/TiO_2_ nanotube. It can be seen that the CuInS_2_ nanoparticles are deposited on the surface of the nanotube and form a CuInS_2_ thin film, which is consistent with the reported results [21]. By comparing the inner diameters of TiO_2_ nanotube and CuInS_2_/TiO_2_ nanotube, it can be obtained that the thickness of the CuInS_2_ thin film is about 10 nm. Figure 2e, f shows the top-view images of the CdS(10)/CuInS_2_/DTTO and CdS(15)/CuInS_2_/DTTO films, respectively. For both films, it can be clearly seen that CdS QDs have been deposited on the TNTs. Furthermore, by comparing Fig. 2e with Fig. 2f, it can be found that more CdS QDs are deposited onto the surface of the CdS(15)/CuInS_2_/DTTO film after 15 CBD cycles, indicating that the deposition amount of CdS QDs increases with the cycle number *n*.

**Fig. 3:**
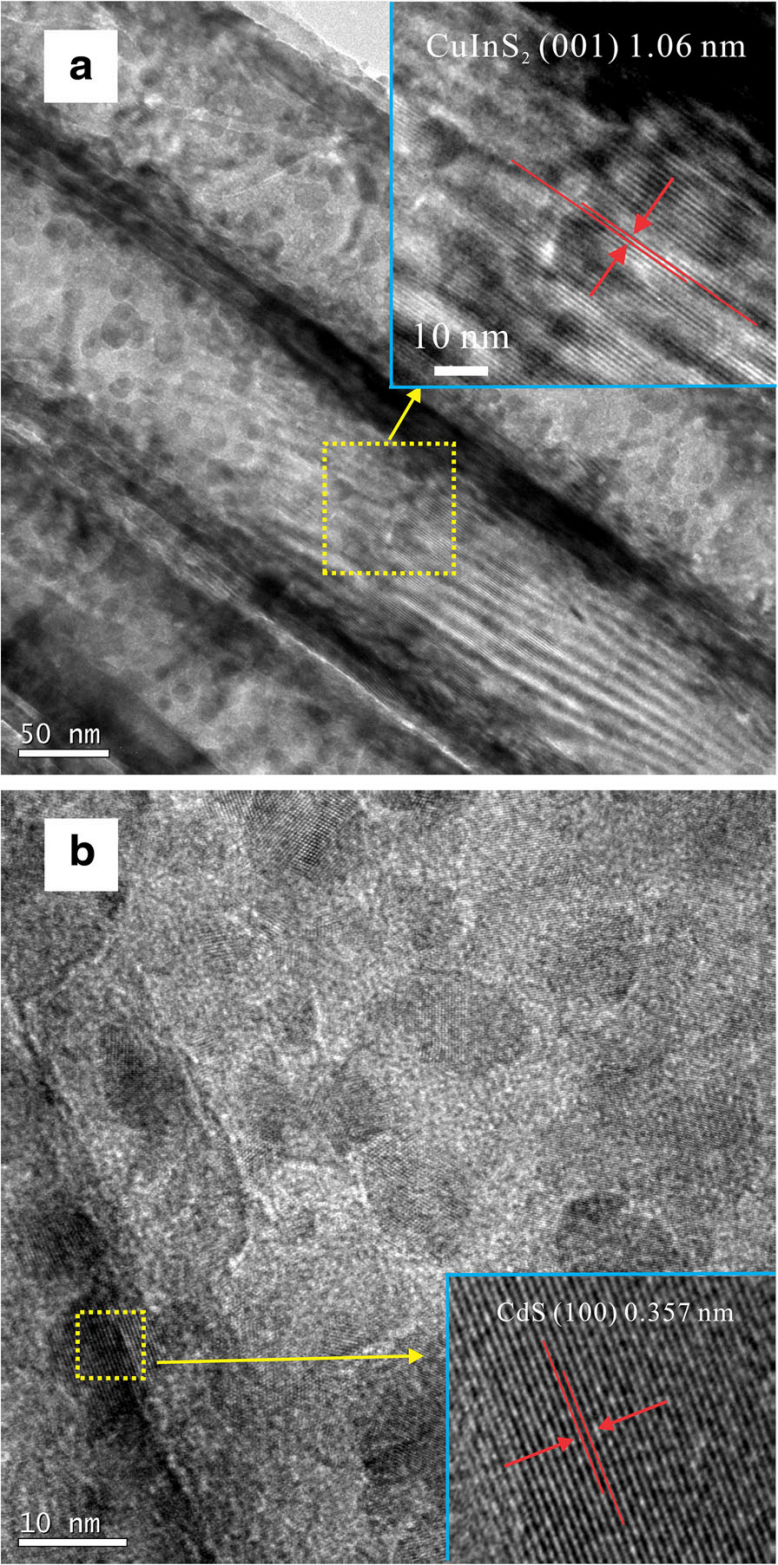
**a** Low- and **b** high-magnification TEM images of the CdS(5)/CuInS_2_/DTTO

Figure 3a, b shows the low- and high-magnification TEM images of the CdS(5)/CuInS_2_/DTTO film, respectively. As shown in Fig. 3a, b, the CdS QDs deposited onto the inner wall of the TiO_2_ tube are observed. The average size of the CdS QDs is about 10 nm. Moreover, as shown in the inset of Fig. 3a, the parallel lattice fringes in the wall of CuInS_2_/TiO_2_ nanotube are observed. After careful measurement, the interplanar spacing of these lattice fringes is 1.06 nm, corresponding to the (001) plane of tetragonal CuInS_2_ (JCPDS 38-0777). The inset of Fig. 3b shows a high-resolution transmission electron microscopy (HRTEM) image of the CdS QDs in the nanotube. The measured lattice spacing for observed fringes is 0.357 nm, which corresponds to the (100) lattice planes of hexagonal CdS (JCPDS 80-0006).

**Fig. 4:**
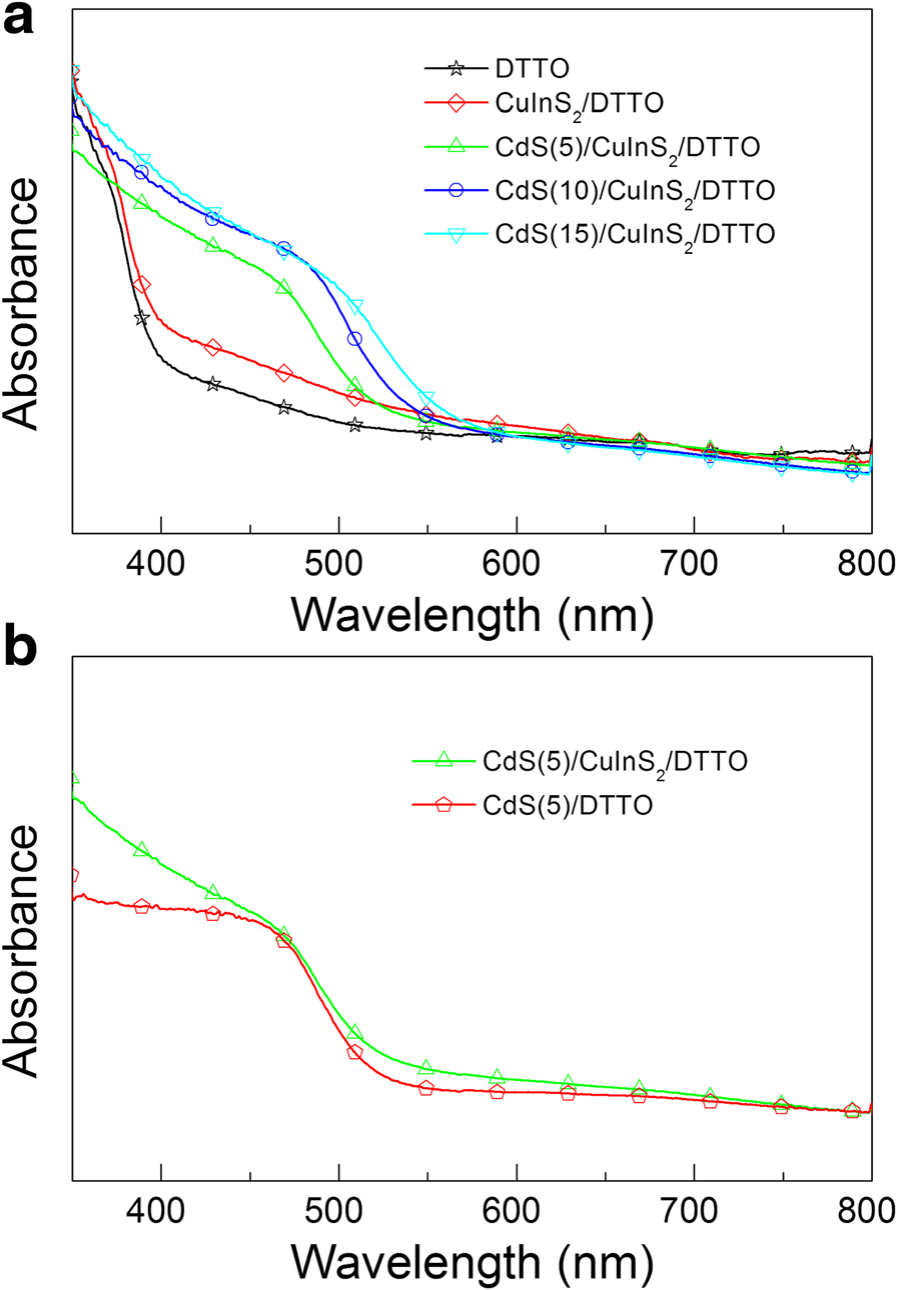
**a** UV–Vis spectra of the DTTO, CuInS_2_/DTTO, CdS(*n*)/CuInS_2_/DTTO (*n* = 5, 10, and 15). **b** Absorption spectrum of the double-sided CdS(5)/DTTO and CdS(5)/CuInS_2_/DTTO films

Figure 4a shows the UV–vis spectra of the DTTO, CuInS_2_/DTTO, CdS(*n*)/CuInS_2_/DTTO films (*n* = 5, 10, and 15). It can be seen that DTTO film absorbs the light with wavelengths less than 400 nm due to the wide bandgap of TiO_2_ (3.0 eV). While CuInS_2_ are deposited, the absorption spectrum of the CuInS_2_/DTTO film is extended from 400 to 700 nm, which is consistent with the previous result [18]. Compared to the CuInS_2_/DTTO film, the absorbance of the spectra of the CdS(*n*)/CuInS_2_/DTTO film significantly increases in the 375–515 nm wavelength region, which can be attributed to the light absorption of CdS. Furthermore, the absorbance of the CdS(*n*)/CuInS_2_/DTTO increases with an increase in CBD cycles, which is mainly due to the increased deposition amount of CdS. These results are similar to those reported in CdS-sentized TNTs [20, 22]. To further investigate the effect of the CuInS_2_ on the CdS/DTTO film, the absorption spectra of the CdS(*n*)/DTTO and CdS(*n*)/CuInS_2_/DTTO films were measured and compared. As an example, Fig. 4b shows the absorption spectrum of the CdS(5)/DTTO and CdS(5)/CuInS_2_/DTTO films, which clearly displays that the absorbance of the spectra of the CdS(5)/CuInS_2_/DTTO film are enhanced compared with the CdS(5)/DTTO film. In particular, the deposited CuInS_2_ significantly extended the response of the CdS(5)/DTTO into the 500–700 nm wavelength region [22], confirming that the CuInS_2_ layer can effectively improve the light absorption property of the CdS/DTTO films.

**Fig. 5:**
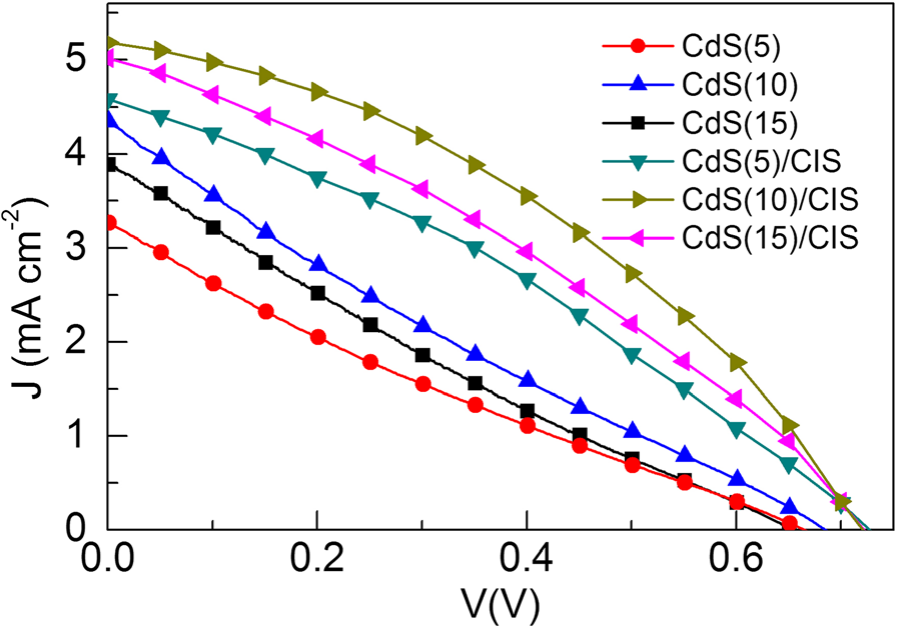
*J–V* characteristics of the double-sided CdS(*n*)/DTTO and CdS(*n*)/CuInS_2_/DTTO electrodes under illumination (*n* = 5, 10, and 15)

Figure 5 shows the *J*–V characteristics of the QDSSCs based on prepared CdS(*n*)/DTTO and CdS(*n*)/CuInS_2_/DTTO electrodes under illumination (*n* = 5, 10, and 15). Four performance parameters for the measured QDSSCs, open-circuit voltage (*V*_oc_), short-circuit photocurrent (*J*_sc_), fill factor (FF), and PCE, have been shown in Table 1.

**Table 1:** Summary of solar cell performances under simulated AM 1.5G solar irradiation

The QDSSCs based on different electrodes	*V*_oc_ (V)	*J*_sc_ (mA cm^−2^)	FF	PCE (%)
CdS(5)/DTTO	0.65	3.27	0.22	0.47
CdS(10)/DTTO	0.66	4.35	0.22	0.65
CdS(15)/DTTO	0.65	3.90	0.21	0.56
CdS(5)/CuInS_2_/DTTO	0.72	4.60	0.32	1.06
CdS(10)/CuInS_2_/DTTO	0.71	5.18	0.38	1.42
CdS(15)/CuInS_2_/DTTO	0.71	5.01	0.33	1.18

For the cells based on the CdS(*n*)/DTTO electrodes, the parameters *J*_sc_ and PCE are increased with the increase of cycle number *n* from 5 to 10 and decreased with the further increase of *n* from 10 to 15. On increasing *n* from 5 to 15, the *J*_sc_ first increases to 4.35 and then decreases to 3.9 mA cm^−2^. This highest *J*_sc_ (i.e., 4.35 mA cm^−2^) is higher than that of CdS-sensitized TiO_2_ nanorod electrode for QDSSCs [23]. Similarly, the PCE first increases from 0.47 to 0.65% and then decreases to 0.56%. The highest PCE of 0.65% is achieved for the cell based on the CdS(10)/DTTO electrode. Moreover, the effects of the cycle number *n* on the values of both *V*_oc_ and FF are not obvious. These results may be explained as follows: As shown in Fig. 4, the amount of CdS loading increases with the increase of *n* from 5 to 10, which helps to strengthen the light absorption and therefore increase the photocurrent. However, as the cycle number *n* increased further (*n* > 10), the electron-transfer resistance in deposited CdS QDs becomes greater as the loading amount of CdS increases (Fig. 2f) and therefore leads to a more serious charge recombination between the photo-generated electrons in CdS and the redox ions in the electrolyte. Therefore, the *J*_sc_, *V*_oc_, and FF may decrease with the further deposition of CdS although the light absorption increases.

**Fig. 6:**
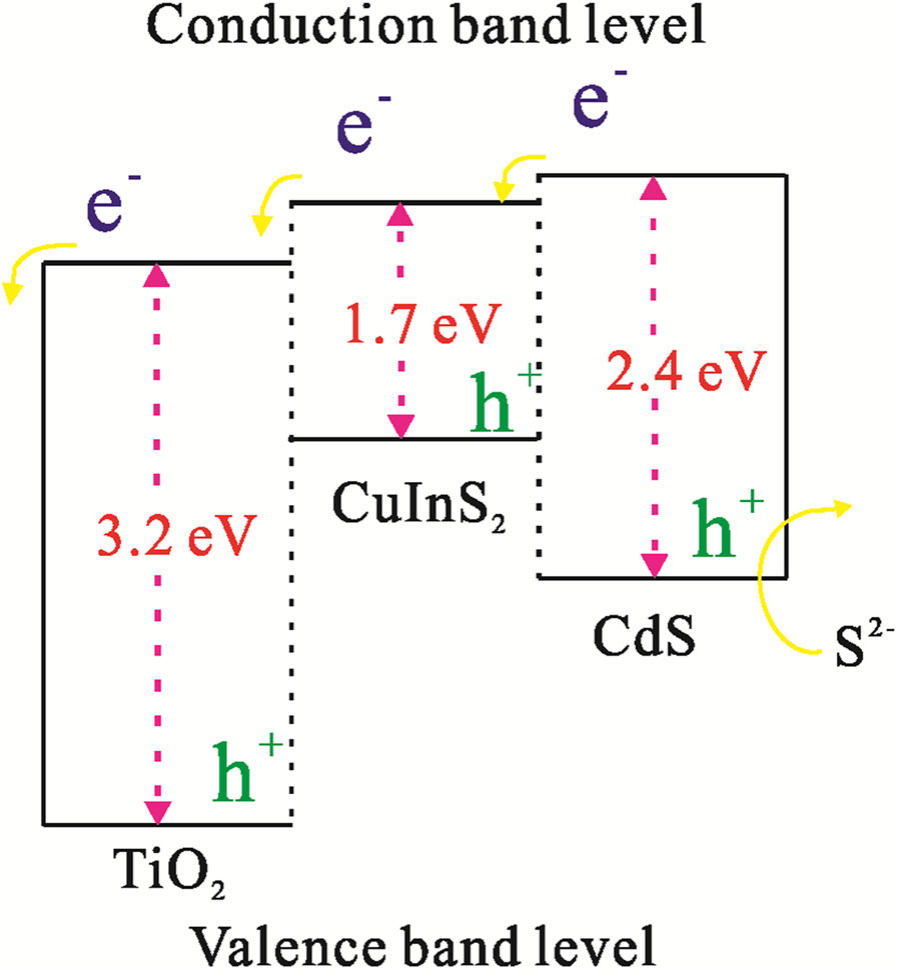
The energy diagram of the CdS/CuInS_2_/DTTO electrode

For the cells based on the CdS(*n*)/CuInS_2_/DTTO electrodes with CuInS_2_ thin film, it can be seen from Fig. 5 that the effect of the cycle number *n* on *V*_oc_ is also not obvious. However, the *V*_oc_ of the cell based on the CdS(*n*)/CuInS_2_/DTTO electrode is significantly higher than that of the cell based on the CdS(*n*)/DTTO electrode, indicating that the CuInS_2_ can effectively reduce the charge recombination. The parameters *J*_sc_, FF, and PCE first increase and then decrease with the increase of *n* from 5 to 15. On increasing *n* from 5 to 10, the *J*_sc_ increases from 4.6 to 5.18 mA cm^−2^, while FF increases from 0.32 to 0.38. As *n* increases further from 10 to 15, the *J*_sc_ and FF decrease to 5.01 mA cm^−2^ and 0.33, respectively. Compared to the cells based on the CdS(*n*)/DTTO electrodes, for a certain cycle *n*, all four parameters *V*_oc_, *J*_sc_, FF, and PCE of the cells based on the CdS(*n*)/CuInS_2_/DTTO electrodes are improved. As shown in Table 1, the highest PCE of 1.42% is obtained for the cell based on the CdS(10)/CuInS_2_/DTTO electrode, which is 2.2 times than that (0.65%) of the cell based on the CdS(10)/ DTTO electrode. Apparently, the PCEs of the cells based on the CdS(*n*)/CuInS_2_/DTTO electrodes have been enhanced largely by the CuInS_2_ layer. On one hand, as shown in Fig. 4, optical absorption of the CdS(*n*)/CuInS_2_/DTTO electrodes was improved in the wavelengths greater than 500 nm compared to the CdS(*n*)/DTTO electrodes, which would lead to an increased photocurrent. On the other hand, the charge recombination may be reduced through the deposited CuInS_2_. For the purpose of facilitating discussion, Fig. 6 shows the energy diagram of the CdS/CuInS_2_/DTTO electrode. As shown in Fig. 6, the conduction energy level of CuInS_2_ lies between that of CdS and that of TiO_2_, which suggests that the photo-generated electrons in CdS can be easily injected into the TiO_2_ through the CuInS_2_ layer. At the same time, it is difficult for the injected electrons in TiO_2_ to recombine with redox ions in the electrolyte because there exists an energy barrier at the interface between the TiO_2_ and CuInS_2_, which leads to a reduced charge recombination in the CdS(*n*)/CuInS_2_/DTTO electrodes and therefore enhances the *V*_oc_, *J*_sc_, and FF.

## Conclusions

In conclusion, the CdS/CuInS_2_ quantum dots-sensitized double-sided transparent TiO_2_ nanotube electrodes are fabricated for the QDSSCs. Our experimental results showed that the deposited CuInS_2_ enhanced the light absorption of the CdS/DTTO electrodes and reduced the charge recombination in the QDSSCs. These two factors resulted in improved photovoltaic performance of the QDSSCs based on the CdS(*n*)/CuInS_2_/DTTO electrodes.

## Abbreviations

DTTO: Double-sided transparent TNT/ITO
FF: Fill factor
*J*_sc_: Short-circuit photocurrent
PCEs: Power conversion efficiencies
QDs: Quantum dots
QDSSCs: Quantum dots-sensitized solar cells
TNT: TiO_2_ nanotube
*V*_oc_: Open-circuit voltage
